# Side-stream products of malting: a neglected source of phytochemicals

**DOI:** 10.1038/s41538-020-00081-0

**Published:** 2020-12-11

**Authors:** Ville M. Koistinen, Marjo Tuomainen, Pekka Lehtinen, Petri Peltola, Seppo Auriola, Karin Jonsson, Kati Hanhineva

**Affiliations:** 1 Institute of Public Health and Clinical Nutrition, University of Eastern Finland, P.O. Box 1627, FI-70211 Kuopio, Finland; 2 Senson Oy Ltd, Niemenkatu 18, P.O. Box 95, FI-15141 Lahti, Finland; 3 School of Pharmacy, University of Eastern Finland, P.O. Box 1627, FI-70211 Kuopio, Finland; 4 Division of Food and Nutrition Science, Department of Biology and Biological Engineering, Chalmers University of Technology, Kemigården 4, SE-412 96 Gothenburg, Sweden; 5 Food Chemistry and Food Development unit, Department of Biochemistry, University of Turku, Turku, Finland

**Keywords:** Nutrition, Metabolomics

## Abstract

Whole grain consumption reduces the risk of several chronic diseases. A major contributor to the effect is the synergistic and additive effect of phytochemicals. Malting is an important technological method to process whole grains; the main product, malted grain, is used mainly for brewing, but the process also yields high amounts of side-stream products, such as rootlet. In this study, we comprehensively determined the phytochemical profile of barley, oats, rye, and wheat in different stages of malting and the subsequent extraction phases to assess the potential of malted products and side-streams as a dietary source of bioactive compounds. Utilizing semi-quantitative LC–MS metabolomics, we annotated 285 phytochemicals from the samples, belonging to more than 13 chemical classes. Malting significantly altered the levels of the compounds, many of which were highly increased in the rootlet. Whole grain cereals and the malting products were found to be a diverse and rich source of phytochemicals, highlighting the value of these whole foods as a staple. The characterization of phytochemicals from the 24 different sample types revealed previously unknown existence of some of the compound classes in certain species. The rootlet deserves more attention in human nutrition, rather than its current use mainly as feed, to benefit from its high content of bioactive components.

## Introduction

Increasing epidemiological evidence is supporting the protective effect of whole grain consumption (but not refined grain mainly constituting of the endosperm) against several chronic diseases and all-cause mortality^1,2^. The bran and germ fraction of a cereal grain are particularly abundant in dietary fiber and vitamins, minerals and thousands of different phytochemicals, recently coined as the ‘dark matter’ of nutrition^3^, all of which may contribute to the beneficial metabolic effects of diets rich in whole grain. As commonly hypothesized, synergistic and additive effects of the various bioactive compounds are mediated through complex endogenic metabolic pathways, facilitating the maintenance of good health^4–7^. Whole grains are rich in several types of phytochemicals, including alkylresorcinols, benzoxazinoids, betaines, flavonoids, lignans, phenolic acids, phytosterols, and tocols, as well as their fatty acid, polyamine, and sugar derivatives, which possess antioxidative and modulatory effects for cellular function and gene expression^5^. However, the exact mechanisms of action remain to be established, because it has proven difficult to link the myriad of biologically active compounds with the health effects on a molecular level; a single compound may not contribute to the effects enough to be even observed.

**Fig. 1:**
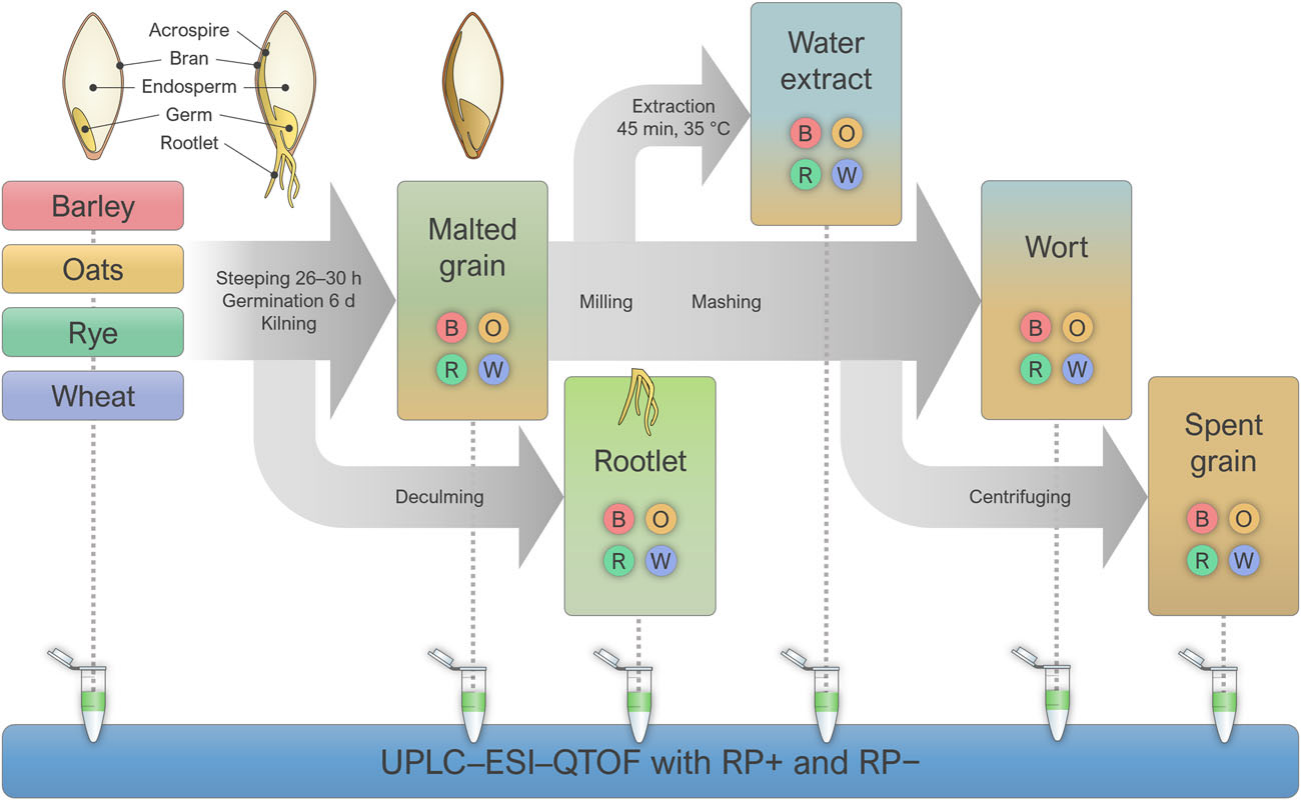
Scheme of the malting, extraction, mashing, and wort separation processes utilized in the study. Intact (native) grains from four cereal species were used throughout the process. The changes in the structure of the grain and the parts used for different stages of the process are illustrated.

Germination represents a crucial developmental stage in plants, inducing several metabolic processes that alter the metabolite profile of the plant remarkably^8^. These changes have been studied in barley^8,9^, rye^10^, and rice^11^, but not extensively on a metabolite level in any cereal species or as a comparison between the species. Germination is utilized in malting, which is a food processing technique where the cereal grain is steeped (immersed in water and drained in cycles), germinated for several days, and dried by kilning. The main side-stream product of the process is the sprout, which consists mainly of rootlets and to a lesser extent the acrospires, which are removed from the dried kernel when the rootlet has clearly appeared (Fig. 1). Malt is most widely used in the brewing of beer and whisky, where the malt undergoes several additional processing steps, including, e.g., mashing, where the malted grains are heated in water, resulting in a hot water extract (wort) and the discarded pellet (spent grain). Barley is by far the most common raw material for malt, although wheat, oats, and rye are also malted in a large industrial scale. Recently, malt has gained interest as a functional ingredient in product development^12^ and bread baking^13–17^. According to these studies, brewer’s spent grains and malts made from oats and sorghum as well as barley rootlets can improve the structure of wheat and gluten-free breads and may increase the nutritional value of these products. Barley malt has also been shown to increase short-chain fatty acid production in rat gut models, suggesting the promotion of colonic health^18,19^. However, knowledge on the effects of malting on individual phytochemicals and the nutritional and health properties of food is limited^12^.

Rootlets removed from the dried malt and spent grains produced after the mashing are a side-stream of the malting process; currently they are being discarded or used primarily as animal feed^16^. The rootlet yield is estimated to be 3 to 5% by weight of the malt^20^; in EU alone, 9.7 million tonnes of barley malt was produced in 2017^21^, which means that 300,000 to 500,000 tonnes of barley rootlets is produced each year in the region. While the usage of rootlets as feed can be justified by avoiding it going to waste, as a rich source of proteins it could cover the yearly protein intake for 4 to 5 million people if it was used directly as food instead. Furthermore, there is some evidence that rootlets have significant antioxidative properties from phytochemicals^22^, which again highlights their nutritional value.

Nontargeted metabolite profiling, especially the application of liquid chromatography–mass spectrometry (LC–MS), is suitable for approaching a research problem involving a large number of chemical compounds belonging to different chemical classes with highly variable concentrations, as in case of whole grain cereals. Instead of measuring single or pre-defined molecules, nontargeted metabolite examination offers a wider, unbiased image of the general phytochemical composition and may reveal unexpected changes in specific metabolites. In agricultural and food science, the approach has applications in determining the metabolic changes induced by e.g., breeding^23^, cultivation conditions^24^, food processing^25,26^, and different geographical origins of the food^27^.

The aim of the current study was to comprehensively determine the phytochemical profile of temperate cereal grains by utilizing nontargeted metabolomics and a combination of databases, scientific literature, and state-of-the-art software to maximize the number of annotated compounds. Another main focus was to examine the effect of malting on the composition and abundance of phytochemicals and whether their levels are different in the side-stream products of malting and brewing, i.e., rootlet and spent grain compared to the intact grain in its original state and the malted grain.

## Results and discussion

### Cereal grain metabolite profiles correlate with genetic relationships of species

**Fig. 2:**
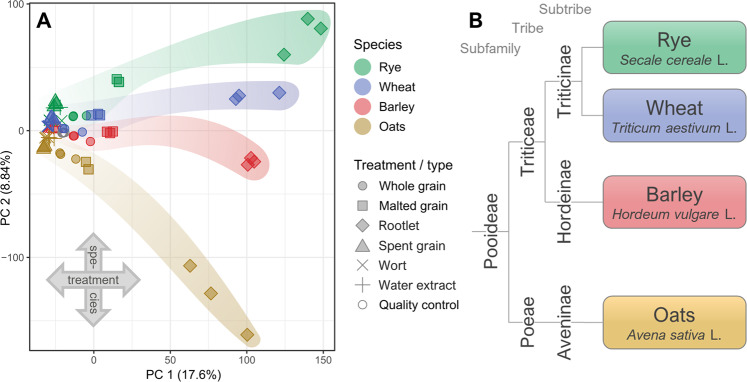
The impact of malting on metabolite profiles of cereals and relation to their phylogeny. **a** Principal component analysis (PCA) of the most intense molecular features (*n* = 12,544, average signal abundance >200,000) in the complete dataset, visualizing the data reduced into the two main orthogonal components explaining the maximal proportion of the variation between samples. Principal component 1 (PC 1) explains 17.6% of the variability between the metabolic profiles of the samples and roughly corresponds with the treatment effect. PC 2 explains 8.84% of the between samples variation and indicates differences between the cereal species. **b** A simplified phylogenetic classification of the four studied temperate cereals, according to Soreng et al.^52^.

A principal component analysis (PCA) was performed on the 12,544 most abundant molecular features (aligned signals detected by the LC–MS instrument; see Materials and methods) from both modes to determine and visualize the differences between the metabolite profiles of each sample (Fig. 2a). The PCA shows the two orthogonal principal components most extensively explaining the variation between samples, PC 1 and PC 2, explaining 18% and 9% of the differences, respectively. PC 1 is strongly related to the effect of malting, with the rootlet samples being very distinct from the rest of the sample groups. On the other hand, PC 2 separates the samples by cereal species, oats being more distant from the other cereals especiallyafter germination. The way the cereal species are separated in the PCA is in line with the genetic relationships between the cereals: in terms of botanical classification, rye and wheat belong to subtribe Triticineae and barley to subtribe Hordeineae within tribe Triticeae, while oats is located in another tribe, Poeae, under subtribe Aveninae (Fig. 2b). Similar connection between the metabolite profiles and genetic relationships have been observed in strawberry, where genetically related cultivars were located close to each other in the hierarchical clustering of metabolites^23^. In a case study conducted on a subfamily of Amaryllidaceae (the amaryllis family of monocot plants), a significant correlation was observed between phylogeny and the chemical diversity and bioactivity of alkaloids^28^. We investigated the PCA further into the third and fourth most explanatory principal components (Supplementary Fig. 1). The main separation occurred again among the rootlet samples of barley, wheat, and rye, with barley rootlet being most distant from all the other samples.

### Whole grains from different species have a diverse and unique phytochemical profile

**Fig. 3:**
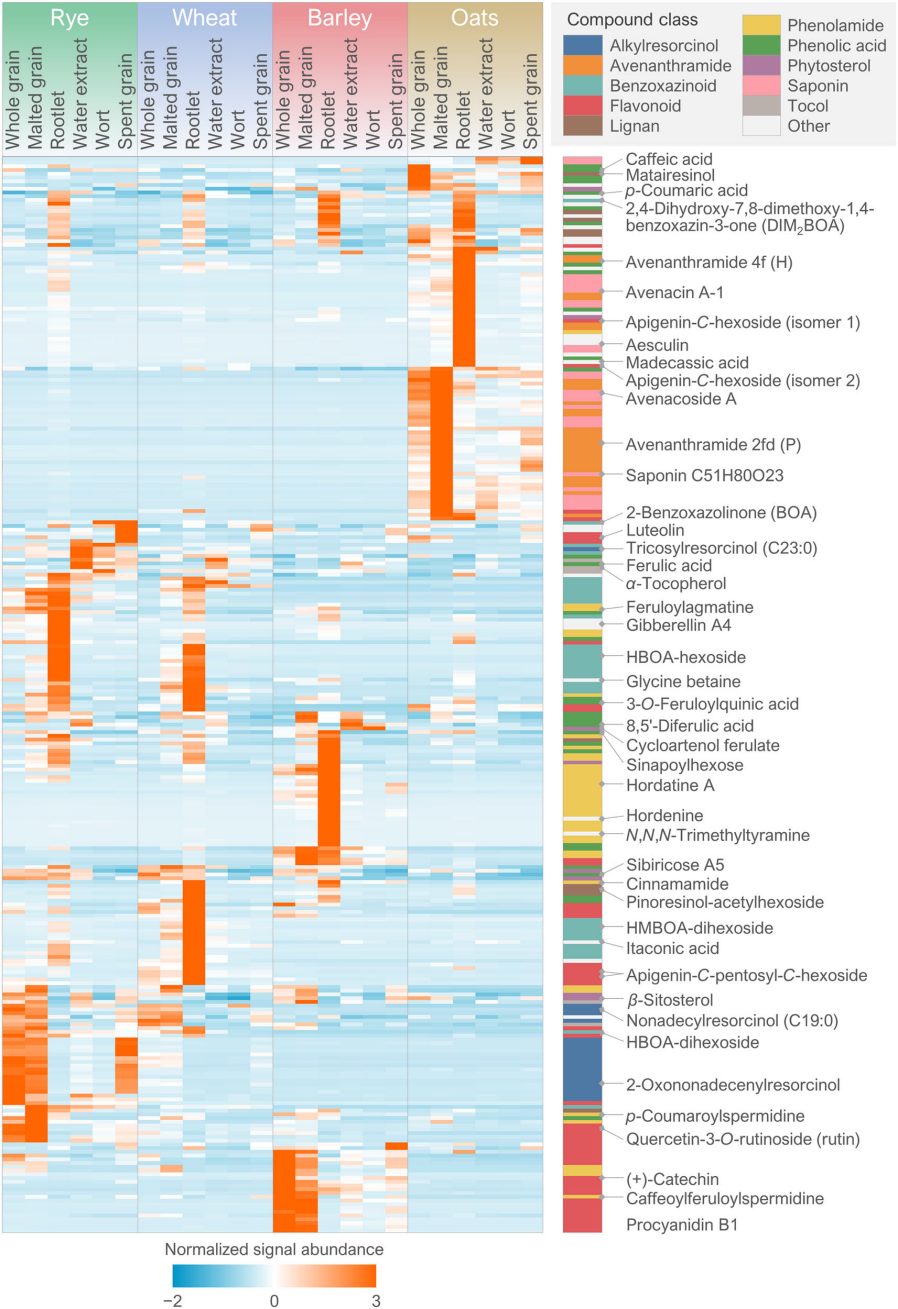
Normalized phytochemical levels during malting. A heat map of the normalized signal intensities (as group averages) of all the annotated phytochemicals (*n* = 285) in all the studied sample types. The chemical classification of each compound is given as a color code. Examples of individual phytochemicals belonging to the various classes, including those discussed in this paper, are highlighted from the heat map.

**Fig. 4:**
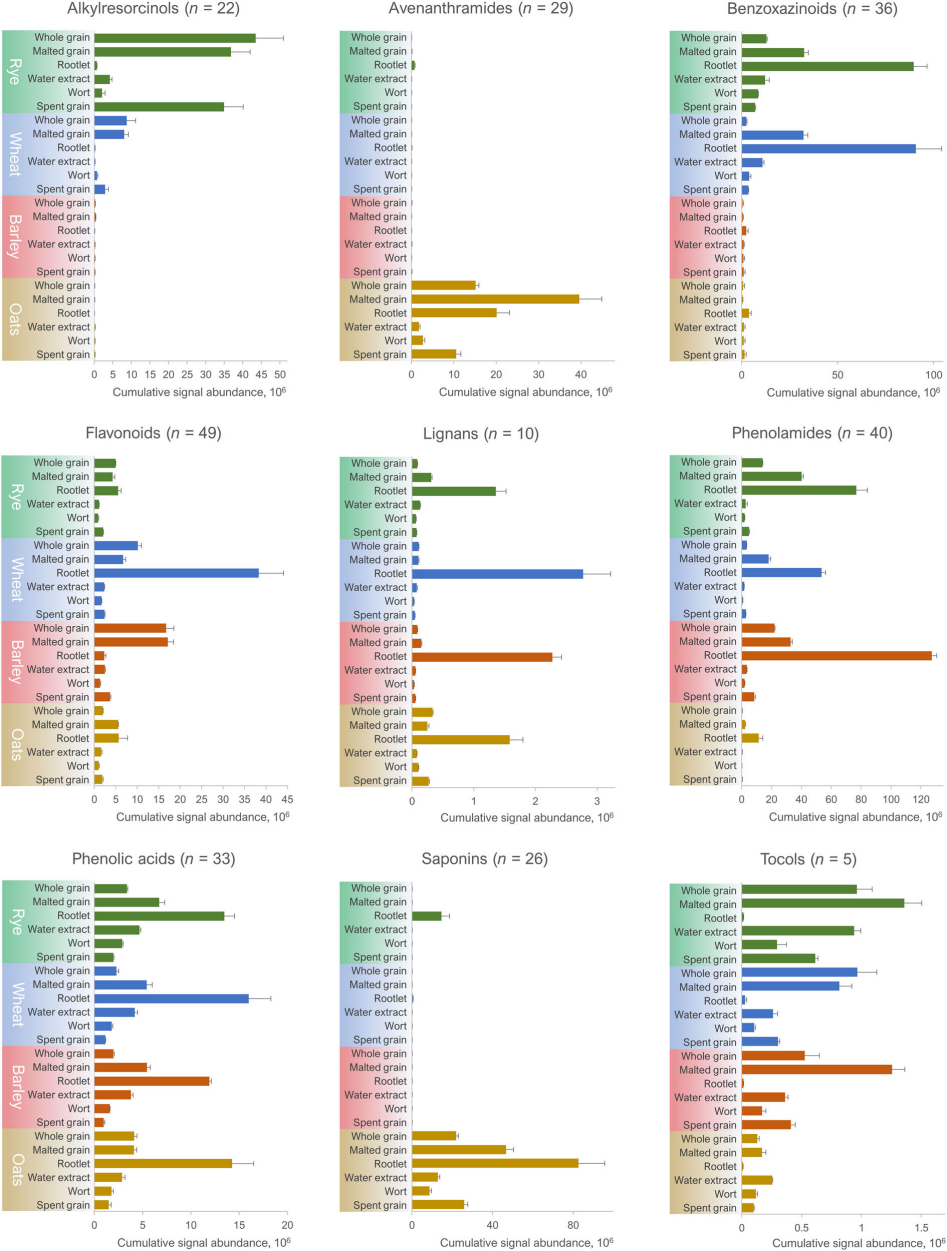
Cumulative abundances of phytochemical classes in the studied samples. The relative abundance of nine phytochemical classes (as cumulative signal abundance of each annotated compound) during the malting process and in the side products (rootlet and spent grain) of four cereal species. The error bars correspond to one standard deviation within the three replicates per sample group. The abundances from water extract and wort are based on wet weight.

We identified and putatively annotated 285 phytochemicals in the samples consisting of four cereal species and six different types of samples, including intact whole grains, malted samples and their water extracts, and the side-stream products (spent grain and rootlets). Figure 3 shows the annotated phytochemicals in a heatmap, arranged with hierarchical clustering to group the compounds based on their abundance across all samples. The compounds belong to more than 13 different chemical classes (Table 1). In terms of the number of individual compounds, flavonoids were the most abundant class with 49 different compounds annotated across all samples, followed by phenolamides (*n* = 40), benzoxazinoids (*n* = 36), and phenolic acids (*n* = 33). Some of the compound classes were specific to certain cereals, such as alkylresorcinols and benzoxazinoids in rye and wheat, and avenanthramides and saponins in oats. However, they were not exclusive to these species: several alkylresorcinols were detected in barley rootlet with nonadecylresorcinol (alkylresorcinol C19:0) being the predominant one; DIMBOA-dihexoside was the benzoxazinoid with the highest levels in oats rootlet and avenanthramide 2pd (O) the main avenanthramide in rye rootlet. However, these compounds were found in lower levels than in those species where they are mainly found. To our knowledge, this is the first time avenanthramides are reported from any other species than oats, suggesting that the synthesis pathway for avenanthramides evolved before oats diverged from the other cereals. Furthermore, benzoxazinoids are herein reported for the first time in oats. Rye rootlet also contains a considerable amount of saponins, consisting of triterpene glycosides (Fig. 4), which among cereals have previously been reported only from oats, millet, and sorghum^29,30^. Several previously uncharacterized saponins were found in oats in addition to the previously known avenacins and avenacosides. However, because of limited reference data currently available, their identity could not be determined beyond compound class and molecular formula in this study. While the cereal species did not differ greatly in the cumulative abundance of flavonoids and phenolic acids, there were significant differences in the compound-level distribution. Within these two classes, each species has a unique set of abundant compounds, such as proanthocyanidins and sinapoylhexose in barley, apigenin-*C*-hexosyl-*O*-pentoside and benzoic acid in oats, chrysoeriol-*O*-neohesperidoside-hexoside and ferulic acid in rye, and apigenin-*C*-pentosyl-*C*-hexoside and 3-*O*-feruloylquinic acid in wheat. Supplementary Table 1 provides a comprehensive list of the phytochemicals annotated in this study, with their abundance and identification details, including observed *m/z*, retention time, and MS/MS fragmentation.

**Table 1:** The number of annotated compounds, categorized by compound class and level of identification according to Sumner et al.^51^: identified with reference standard (level 1), putatively annotated based on publicly available MS/MS data (level 2), and putatively characterized compound class based on physicochemical characteristics (level 3). The sample type with the highest cumulative abundance is listed for each class. *Other compound classes include alcohols (*n* = 1), alkaloids (*n* = 2), betaines (*n* = 2), diterpenoids (*n* = 2), esters (*n* = 1), sphingolipids (*n* = 1), and triterpenoids (*n* = 1).

Compound class	Level 1	Level 2	Level 3	Total	Highest abundance
Alkylresorcinols	1	16	5	22	Rye, whole grain
Avenanthramides	—	28	1	29	Oats, malted grain
Benzoxazinoids	—	36	—	36	Rye & wheat, rootlet
Coumarins	—	4	—	4	Oats, rootlet
Dicarboxylic acids	3	3	1	7	Wheat, rootlet
Flavonoids	1	41	7	49	Wheat, rootlet
Lignans	—	9	1	10	Wheat, rootlet
Phenolamides	—	40	—	40	Barley, rootlet
Phenolic acids	9	22	2	33	Oats, rye & wheat, rootlet
Phenolic aldehydes	2	4	—	6	Oats, rootlet
Phytosterols	—	7	1	8	Rye, whole grain
Saponins	—	10	16	26	Oats, rootlet
Tocols	—	5	—	5	Rye & barley, malted grain
Others*	—	9	1	10	Barley, rootlet
Total	16	234	35	285

**Fig. 5:**
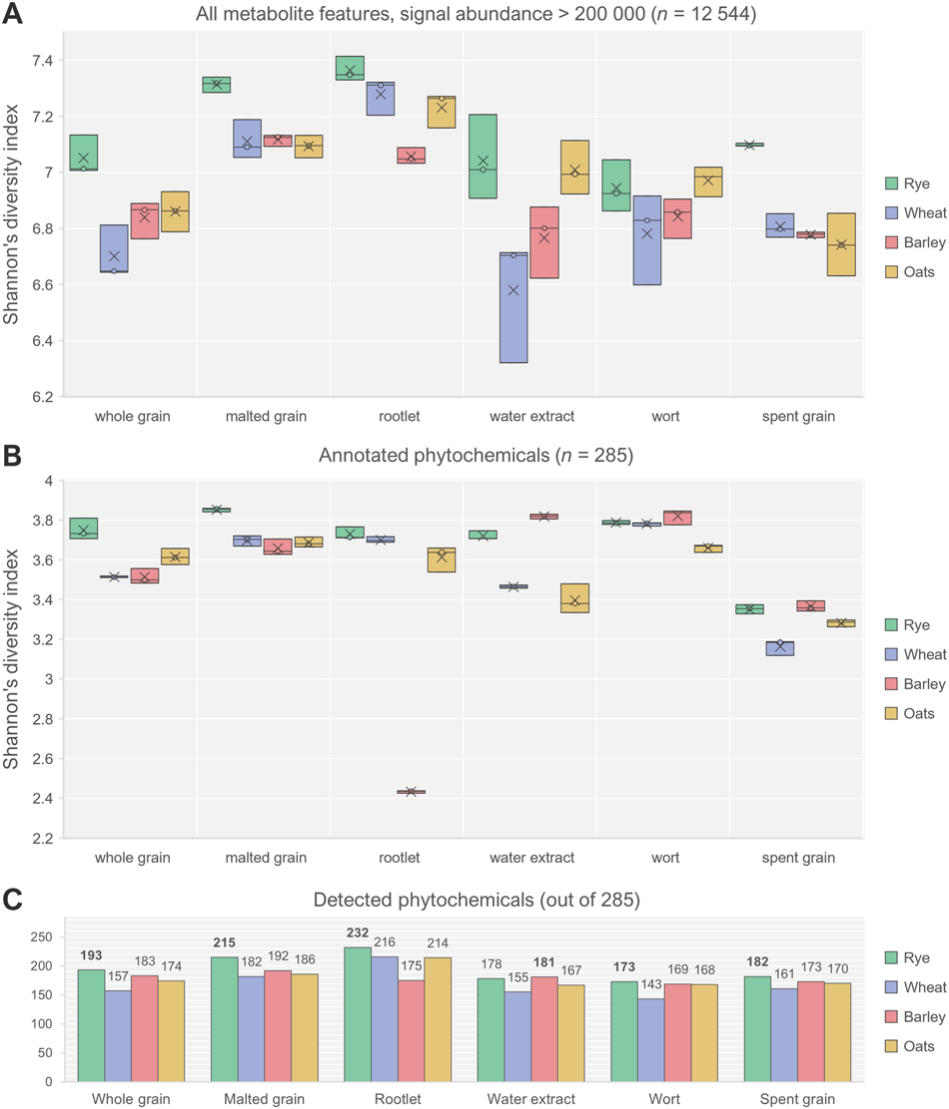
The metabolite and phytochemical diversity of the studied cereal samples. **a** Shannon’s diversity index of all the detected metabolite features with signal abundance over 200,000 counts (box plot with first and third quartiles, average [×] and median [○] values). **b** Shannon’s diversity index of all the annotated phytochemicals. **c** The richness (number of detected phytochemicals) in each sample type (signal-to-noise ratio > 5 considered as limit of detection).

According to the prevalent hypothesis, dietary phytochemicals have synergistic and additive bioactivity^4^; thus, a wider range of phytochemicals in a food may further increase their contribution to the health-promoting properties of whole plant foods compared to more refined foods with a narrower range of bioactive compounds. Therefore, we also assessed the overall metabolite and phytochemical diversity of the samples with Shannon’s diversity index and the number of detected phytochemicals. Figure 5a shows the overall metabolite diversity of the samples: in the intact whole grain, malted grain, rootlet, and spent grain, rye has the highest diversity compared to the other species, with rye rootlet having the highest diversity index of all samples. A similar trend can be observed when looking at the diversity only within those metabolites annotated as phytochemicals (Fig. 5b). In barley, the phytochemical diversity is considerably lower in the rootlet compared to rootlets of the other cereals. This may be attributed to few individual compounds, such as hordenine (a barley alkaloid), which showed a 150-fold increase in abundance in barley rootlet compared to whole grain (on dry weight basis). Its high abundance compared to other phytochemicals detected in barley rootlet lowers the diversity index. Barley rootlet was also clearly separated from the other samples by the fourth principal component in the PCA (Supplementary Fig. 1). The bigger change in the metabolite and phytochemical diversity of barley compared to the other cereals may also be related to the genetic differences, some of which may exist because most cultivars of barley, unlike the other species, has been cultivated and bred mainly for malting and brewing purposes for several millennia. This hypothesis may be supported by previous identification of hordenine as a selective dopamine D_2_ receptor agonist, potentially contributing to the rewarding effect of drinking beer^31^; nevertheless, the hypothesis remains speculative and requires further research.

To determine the richness of phytochemicals, we counted the number of different detected phytochemicals in each sample type, using signal-to-noise ratio above 5 as the threshold for a true detection of the compound (Fig. 5c). Rye rootlet had the highest number of annotated phytochemicals, 232, which accounts for more than 80% of all the phytochemicals annotated in this study. Rootlets had the highest number of phytochemicals out of the oat and wheat fractions, respectively, as well; while in barley, malted grain had the highest number. The lowest number of phytochemicals (*n* = 143), out of all samples, was detected in wheat wort. Within intact whole grains, rye was also the richest source of distinct phytochemicals (*n* = 193). While it can be hypothesized that higher diversity and richness of phytochemicals adds to the health-promoting potential of the food, we are still far from understanding the complex mechanisms behind the health effects and the specific contributions from each compound^3^.

### Malting significantly alters the phytochemical composition

Statistically significant changes occurred in the abundance of all the 285 annotated phytochemicals during malting, when comparing whole grain with malted grain or rootlet in the four studied cereals (pairwise *t-*test, FDR-corrected *p*-value <0.10; see Supplementary Table 1).

The compound classes responded in a different manner to malting. The cumulative levels of alkylresorcinols remained nearly the same in malted grain and spent grain of rye and wheat compared to native whole grain, with a small portion present in the extracted samples (water extract and wort) (Fig. 4). However, they were nearly absent from the wheat rootlets and had very low levels in the rye rootlets as well. This is in contrast to rice, where alkylresorcinols are mainly found in the seedlings but not in the grains, which also contain less fiber compared to the temperate whole grains^32^. In oats, the cumulative levels of avenanthramides increased by 2.6-fold in the malted grain compared to intact whole grain. Up to 25-fold increase has been reported previously after a slightly longer germination^33^. While other grains than oats contained negligible levels of avenanthramides, they were present in rye rootlet, mainly as avenanthramide 2pd / O (Fig. 4). Malting significantly increased the levels of benzoxazinoids, especially in wheat, by 13-fold in malted grain and by 37-fold in rootlet compared to intact grain (on dry weight basis). The high increase of benzoxazinoids in the rootlet can be explained by the role of benzoxazinoids as defence molecules, which the plant produces against pests and diseases not only into its above-ground parts but also into the rhizosphere^34^. A similarly high increase in the rootlet levels was also observed for lignans in all studied cereals (Fig. 4); although the role of lignans in plants is not yet fully established, it is likely related to defence as well^35^. Unexpectedly, the most abundant lignan found in this study was a novel compound in cereals, pinoresinol acetylhexoside, which had high levels in the rootlet samples of all studied cereal species. Wheat rootlet had 5 to 6-fold higher levels of the compound compared to other rootlet samples, while only traces were observed in the other sample types (Supplementary Table 1). Pinoresinol acetylhexoside has been previously characterized only from globe artichoke^36^. The tentative (level 2) identification and fragmentation of pinoresinol acetylhexoside is shown in Supplementary Fig. 2.

The flavonoids in each cereal species responded very differently to the malting process, which may be explained by the different flavonoid synthesis pathways between the species, resulting in a unique flavonoid profile. In oats, the cumulative flavonoid levels were nearly 3-fold higher in malted grain and rootlet compared to whole grain (Fig. 4). In wheat, a minor decrease in the cumulative levels was observed for the malted grain, while the levels increased by 3.8-fold in rootlet compared to whole grain. This increase was attributed mainly to the *de novo* synthesis of two isomers of apigenin-*C*-pentosyl-*C*-hexoside; together, the two flavone glycosides accounted for over 75% of the combined abundance of flavonoids detected in wheat rootlet. In barley, the absence of proanthocyanidins in the rootlet resulted in significantly lower (7-fold) abundance of total flavonoids compared to whole grain (Supplementary Fig. 3).

The overall levels of phenolic acids were affected quite similarly by malting: they increased about 2-fold in malted grain (except for oats, where the levels remained the same) and 3 to 7-fold in rootlet in comparison to whole grain (Fig. 4). The predominant phenolic acid in the rootlets of oats, rye, and wheat was 3-*O*-feruloylquinic acid, one of the chlorogenic acids postulated to mediate the beneficial health effects of coffee^37^. Phenolamides, which are polyamine derivatives of phenolic acids, behaved in a similar fashion to their precursors: their cumulative levels increased in both malted grain (from 1.5-fold in barley to 11-fold in oats) and rootlet (from 6-fold in rye to 50-fold in oats). Barley rootlet was particularly abundant in caffeoyl-, sinapoyl- and feruloylagmatine (Supplementary Table 1). Previously, phenolamides have been observed to increase in sourdough fermentation of wheat bran^38^; they were also characterized from barley and beer by Pihlava^39^. Regarding their response to malting in this study, saponins can be divided into two groups: one containing all the avenacosides, having its highest abundance in malted grain, while the other one, containing avenacins, is highly increased in the rootlet via biosynthesis (Supplementary Fig. 3). Indeed, avenacins are known to inhibit the growth of fungi in the rhizosphere of oats^40^. Malting increased the levels of tocols (vitamin E) in barley and rye malted grain compared to native grain; however, their levels in the rootlet were low in all cereals, likely due to the naturally low fat content of the rootlet.

All the compound classes were at least to some extent extractable into the water extract (ambient temperature extraction in which cell structures remain mainly intact) and wort (hot water extract from the remaining pellet after water extraction, causing cell structure hydrolysis by endogenous enzymes) (Fig. 4). Thus, they can be expected to be present in the end products, such as beer^41^; however, some individual compounds, such as certain flavonoids, accumulated into the rootlet and were missing from the extracts produced from the malted grain, from which the rootlet was already removed (Supplementary Table 1). Hydrophilic semi-polar compound classes, such as benzoxazinoids and phenolic acids, but also relatively non-polar saponins (likely because of the sugar decorations) and tocols, were relatively well extracted by water. In contrast, lipophilic alkylresorcinols and certain semi-polar classes, such as avenanthramides and phenolamides, had much lower extractability into the water extract and wort. The further solubilization of cereal components caused by mashing allowed more compounds to be extracted. Nevertheless, the remaining spent grain still contained significant amounts of alkylresorcinols and avenanthramides.

A major limitation in an nontargeted LC–MS metabolomics study, although being the most powerful method for a wide-scale characterization of compounds, is the large proportion of unknowns, partially because of the extremely high number of detected signals, exceeding 100 000 in this study. Plants can synthetize up to hundreds of thousands of secondary metabolites, and the current spectral databases only contain a fraction of them to allow identification. Although several more compounds were possible to be annotated based on existing literature, the compounds found in this study thus do not represent the complete range of phytochemicals existing in cereals. The unknowns, many detected as high-intensity signals, also pose potential bias to the number of detected phytochemicals, because the different plant species may not have been studied equally thoroughly regarding their phytochemical content. However, the cereals studied in the current work are closely enough related that many of the phytochemicals were detected in all of them. All of the four species are also widely studied, and we have previously reviewed the current knowledge on the phytochemicals present in them^42^. Regarding the interpretation of the results, an ongoing challenge is to elucidate the complex mechanisms by which phytochemicals may benefit health and what is the dose required for clinically relevant effects – as far as the current evidence goes for foods rich in phytochemicals, such as whole grain, more is better^2^. Worth noting is that the phytochemical content may vary depending on the conditions used during *e.g*. germination and kilning, such as the time and temperature. The current level of knowledge also does not properly allow to compare the metabolite or phytochemical diversity of whole grain to other plant-based foods, because phytochemicals have not been extensively characterized from foods with nontargeted methods. Although it can be safely assumed that in whole grains the pool of phytochemicals works towards beneficial health effects, it cannot be ruled out that some individual compounds would have undesirable effects when consumed separately.

We describe here one of the most comprehensive efforts thus far in characterizing the phytochemical profile of a single food group, namely cereals and products from their food processing, such as malt and rootlet. It was shown that whole grains and their malted products indeed contain a wide range of bioactive compounds—recently coined as the ‘dark matter’^3^ of plant foods. Because of the suggested health benefits from the phytochemicals working in synergy, they deserve more attention to further develop analytical methods and spectral databases for more extensive characterization of the compounds. This in turn can be followed by deciphering the mechanisms of action and dose–response relationships and promoting healthy and sustainable plant-based diets.

The side-stream products of malting, particularly rootlet, is currently treated as animal feed. Instead of ending up in the final products (*e.g*., malt and beer), a substantial portion of the phytochemicals end up in the side streams, emphasizing the great potential of these fractions to be recovered and used for healthy, nutritious foods for humans. Rootlets are being increasingly investigated to overcome their bitter taste and to unleash their potential to fortify food products, such as bread. Adding the fact that the side-stream products produced in high quantity are also rich in protein, their nutritional value may be too high to justify their usage as feed rather than food in the current global food environment, struggling for sustainability and food security.

## Methods

The methods were performed in accordance with relevant guidelines and regulations and approved by the Faculty of Health Sciences, University of Eastern Finland.

### Malting of cereal grains

Whole grains of two-row barley (*Hordeum vulgare* L. var. Harbinger), oats (*Avena sativa* L. var. Steinar), winter rye (*Secale cereale* L. var. Reetta), and common wheat, spring variety (*Triticum aestivum* L. var. Amaretto), all cultivated in Finland, were used in the malting process. The grains were steeped for 26 to 30 h with a wet–dry–wet steeping program; barley and wheat were wet steeped for 6 h and oats and rye for 4 h (both at 13 °C) before and after 18 h of dry steeping at 15 °C. All grains were germinated for 6 days at 15 °C, after which they were dried with a gentle kilning program (designed for pilsner malt) to a final temperature of 83 °C and final moisture of 4%. The rootlets were separated from the malt after drying. Water extract was produced by mixing ground malt with water and incubating under agitation for 45 min at 35 °C. The liquid fraction (water extract) was separated by centrifugation. The wort, a combination of water extraction by temperature gradient and hydrolysis of cell structures by endogenous enzymes in malts, was produced from the pellet by adding more water and performing a standard step infusion mashing with steps at 52 °C (20 min), 64 °C (30 min), 71 °C (20 min), and 81 °C (20 min). After cooling to room temperature, the mixture was centrifuged to produce the wort sample (supernatant) and the spent grains sample (pellet). The samples (three replicates of each type) were obtained from the native/intact whole grains, malted grain (without rootlet), wort, and the side-stream (rootlet, water extract of malted grain, and spent grain) (Fig. 5). The moisture content of the samples is specified in Table 2.

**Table 2:** Moisture content (% w/w, average) in the analyzed samples. The moisture content of the rootlets was not measured separately, but it can be assumed equivalent to that in malted grain because the rootlets were separated from the malted grains right after the drying process.

Sample type	Rye	Wheat	Barley	Oats
Intact whole grain	11.9	12.9	12.5	11.4
Malted grain	3.9	4.0	3.8	2.9
Water extract	89.8	93.2	92.0	95.5
Wort	85.5	83.1	84.8	87.2
Spent grain	75.4	71.3	73.0	66.5

### Sample preparation

For the whole grains, malted grains, and rootlets, frozen (−80 °C) samples were homogenized using tissue homogenizer (TissueLyser II, Qiagen) with liquid nitrogen-chilled grinding jars and stainless steel balls. Powdered samples were weighed, and ice-cold 80% MeOH in a ratio of 600 µl of solvent per 100 mg of powder was added. The samples were vortexed (5 s, RT), sonicated (15 min, RT), vortexed again and centrifuged (14 000 rpm, 10 min, +4 °C). After centrifugation, the supernatant was filtrated trough 0.2 μm PTFE filters into HPLC glass vials.

For the spent grain, ice-cold samples were weighed and ice-cold 80% MeOH in a ratio of 600 µl of solvent per 100 mg of sample was added. For the wort and water extract, the ice-cold samples were mixed with ice-cold 100% MeOH in a ratio of 300 µl of solvent per 100 µl of sample. These samples were further processed the same way as the whole grain, malted grain, and rootlet samples, except that the wort and water extract samples were not sonicated. Quality control (QC) was prepared by pooling 20 µl of each sample type after the centrifugation step. The mixture was filtrated through the PTFE filters.

### LC–MS/MS analysis

The LC–MS analysis was performed as described previously by Hanhineva et al.^43^. In brief, the samples were analyzed using liquid chromatography quadrupole time-of-flight mass spectrometry UHPLC–QTOF–MS system (Agilent Technologies), which consisted of a 1290 LC system, a Jetstream electrospray ionization (ESI) source, and a 6540 UHD QTOF mass spectrometer. The samples were separated using reversed-phase (RP) chromatography (Zorbax Eclipse XDB-C18, particle size 1.8 µm, 2.1 × 100 mm; Agilent Technologies). The elution solvents were water (A) and HPLC grade methanol (B), both containing formic acid 0.1% *v/v*. The gradient was as follows for the ratio of solvent B: 0–10 min: 2 → 100% B; 10–14.5 min: 100% B; 14.5–14.51 min: 100 → 2% B; 14.51–16.5 min: 2% B. Data was acquired with both positive and negative polarity. Quality controls were injected at the beginning and at the end of the MS run and after every ten injections. Automatic data-dependent MS/MS analysis was performed on one sample representing each sample type. The sample tray was kept at +4 °C during the analysis. Three replicates for malted grain and rootlet and three technical replicates for whole grains, water extract, spent grain, and wort, were analyzed in a completely randomized order.

### Data analysis

The raw data from the LC–MS instrument was processed in MS-DIAL version 3.90^44^. For the peak picking, MS1 tolerance was set to 0.01 Da, MS2 tolerance 0.025 Da, *m/z* range 50–1500 (small molecules), minimum peak amplitude 2000 signal counts, and mass slice width 0.1 Da. Peak smoothing was performed using linear weighted moving average; the smoothing level was 3 scans and minimum peak width 5 scans. The adduct ions were selected as follows: [M + H]^+^, [M + NH_4_]^+^, [M + Na]^+^, [M + CH_3_OH + H]^+^, [M + K]^+^, [2 M + H]^+^ for the positive mode and [M − H]^−^, [M − H_2_O − H]^−^, [M + Cl]^−^, [M + FA − H]^−^, [2 M − H]^−^ for the negative mode. For the peak alignment, *m/z* tolerance was 0.015 Da and retention time tolerance 0.05 min. *Gap filling by compulsion* function was utilized to forcibly detect peak areas ad hoc within 5 data points even if no local peak maxima were detected. The peaks of the annotated compounds were curated manually if the automated peak picking had resulted in integration errors. After aligning the detected signals across all samples, the remaining 101 546 individual molecular features, including those originating from the positively and negatively ionized molecules, were compiled into an Excel datasheet for further data analysis and compound annotation.

Principal component analysis (PCA) was performed for the 12 544 most abundant molecular features (average peak area >200,000 signal counts) in RStudio v. 1.1.447 utilizing in-house scripts, the *ggplot* function in R package *ggplot2*, and biplot scaling based on Euclidean distance matrix. Quality control samples were plotted in the PCA to assess potential signal drift during the LC–MS run. Shannon’s diversity index for the phytochemical abundances was calculated in R Studio using *vegan* package version 2.5–6^45^ similarly to Marzetz et al.^46^. The heatmap was produced in Multiple Experiment Viewer v4.9.0. For this purpose, the relative abundances were first normalized per each compound based z-normalization: *x* = (*x* − *x*¯_*row*_)/*SD*_*row*_ Benjamini–Hochberg false discovery rate correction was used for the pairwise *t*-test results using an online calculator.

### Annotation of compounds

The molecular features were annotated in a semi-targeted manner, utilizing literature on previously detected phytochemicals in cereals^10,33,42^. A NIST compatible MSP database file^47^, containing e.g., MassBank^48^, GNPS (Global Natural Products Social Molecular Networking)^49^, RIKEN spectral databases, and our in-house reference standard library, was utilized in MS-DIAL for additional annotations and mass spectral comparison. The METLIN database^50^ was used via its online user interface. The reliability of each annotation was assessed according to the Metabolomics Standards Initiative^51^: level 1 was given to true identifications confirmed with a reference standard analyzed with the same instrument and LC–MS method; level 2 included putative annotations based on the exact mass, calculated molecular formula, and MS/MS fragmentation spectra; level 3 was used as the classification for putative characterization of compound class, based on characteristic MS/MS fragmentation pattern and additional physicochemical properties, such as retention time. Pinoresinol acetylhexoside was putatively annotated based on characterization of the MS/MS fragmentation pattern.

### Reporting summary

Further information on research design is available in the Nature Research Reporting Summary linked to this article.

## Supplementary information

Supplementary Figures 1-3

Supplementary Table 1

Reporting summary

## Data Availability

The authors declare that the data supporting the findings presented in this study are available within the paper and its supplementary information files.
