# Supplementary material for: Side-stream products of malting: a neglected source of phytochemicals
Source: NPJ Sci Food. 2020 Dec 11;4:21. doi: 10.1038/s41538-020-00081-0 (PMC7733442; doi:10.1038/s41538-020-00081-0)
Supplement: Supplementary file 1 — Supplementary Figures 1-3 [file 41538_2020_81_MOESM1_ESM.docx]

# Supplementary material


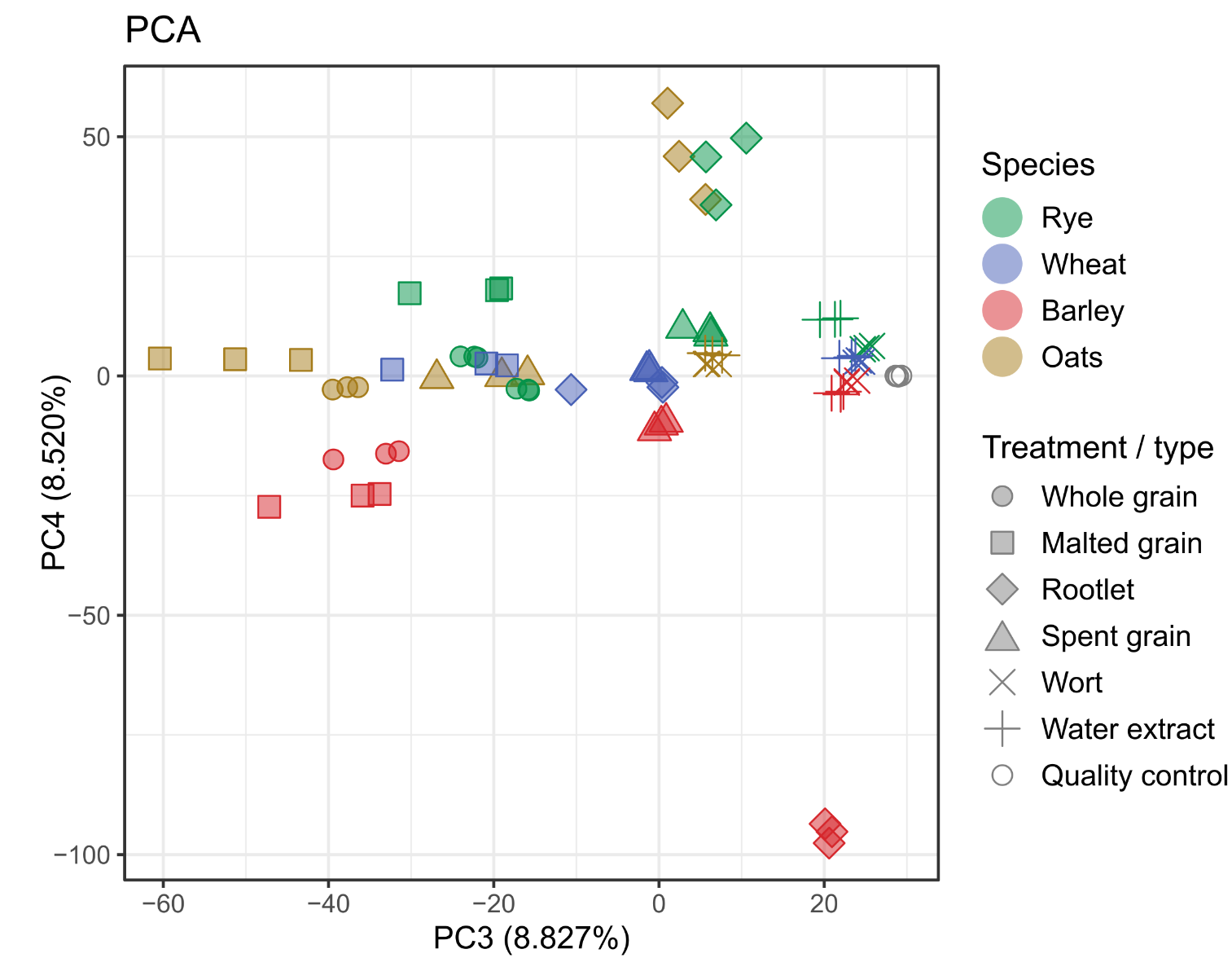


**Supplementary Figure 1.** Principal component analysis (PCA) of the most intense molecular features (*n* = 12 544, average signal abundance > 200 000) in the complete dataset, visualizing the data reduced into the third and fourth most explanatory orthogonal components. Principal component 3 (PC 3) explains 8.8% of the variability between the metabolic profiles of the samples. PC 4 explains 8.5% of the between-samples variation.

**
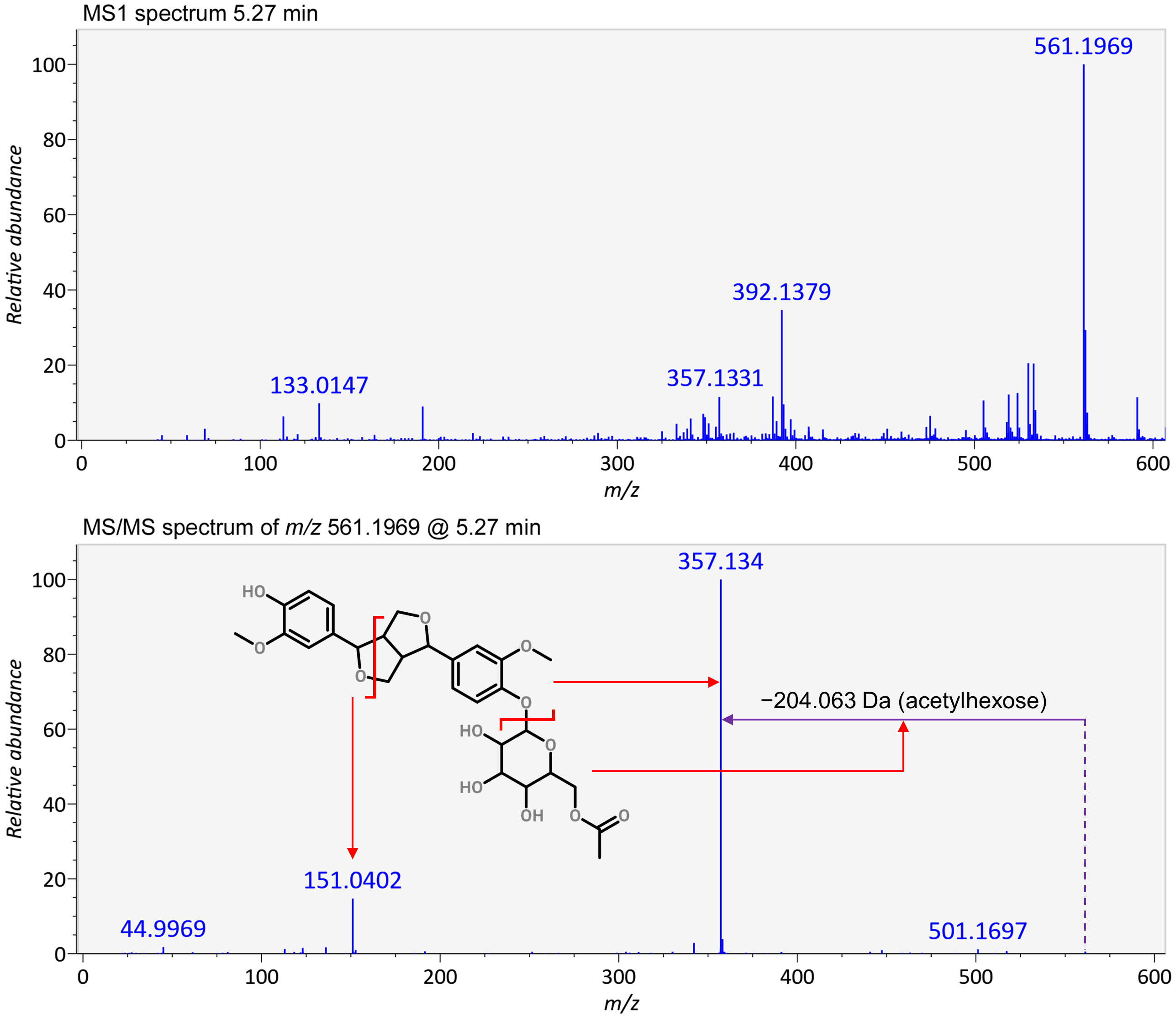
**

**Supplementary Figure 2.** The mass spectra utilized for the structural elucidation of pinoresinol acetylhexoside in the RP negative mode with tentative structure, fragmentation, and the neutral loss of the acetylhexose moiety. The pinoresinol aglycone at *m/z* 357.1331 can be observed already in the MS1 spectra as a smaller in-source fragment peak originating from pinoresinol acetylhexoside (*m/z* 561.1969) and as the main fragment in the MS/MS spectrum.

**Supplementary Figure 3 (below).** Heat maps of the normalized signal intensities (as group averages) of the annotated phytochemicals separately for each compound class (**Figure 3 A–K**) in all the studied sample types.

**A) Alkylresorcinols**


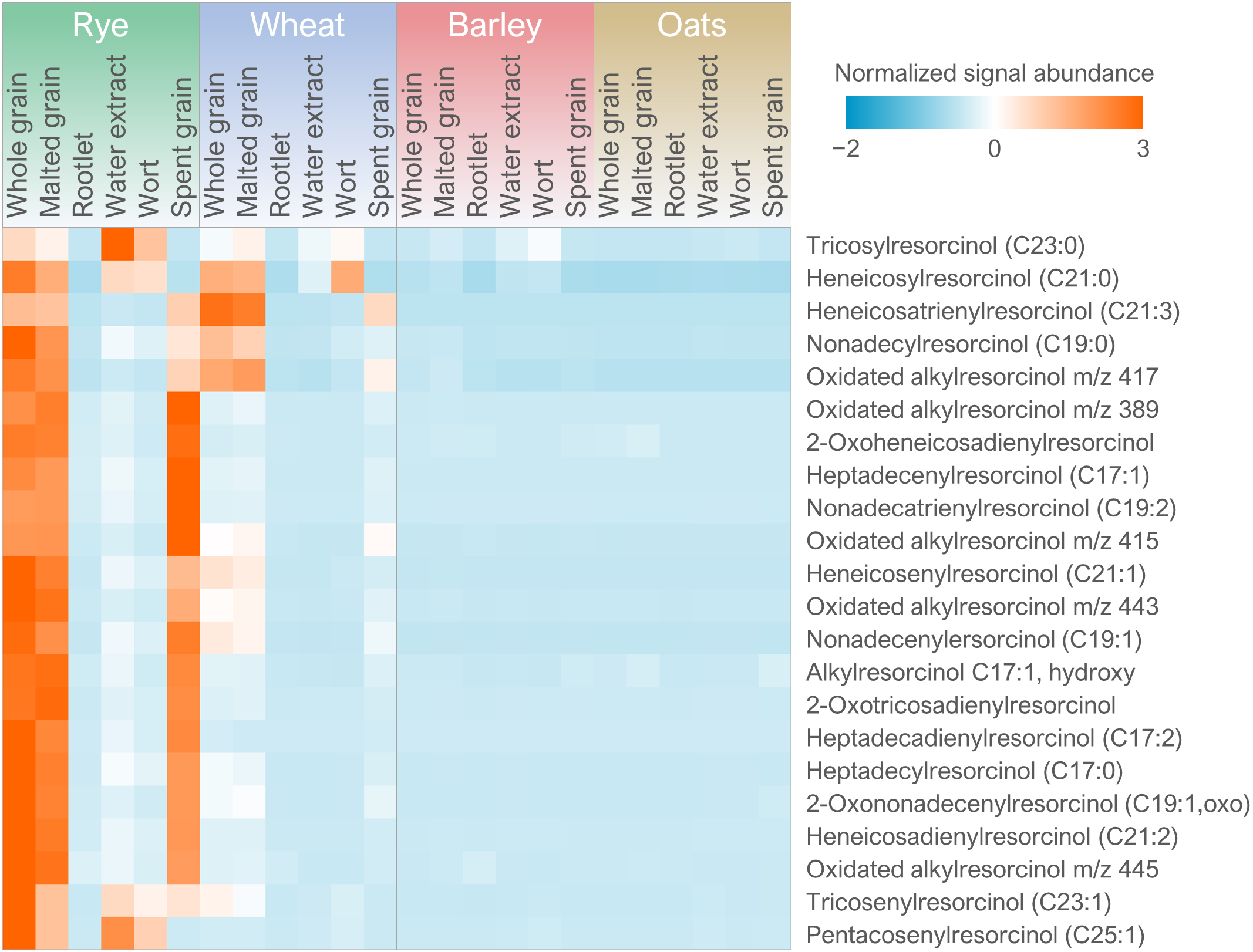


**B) Avenanthramides**


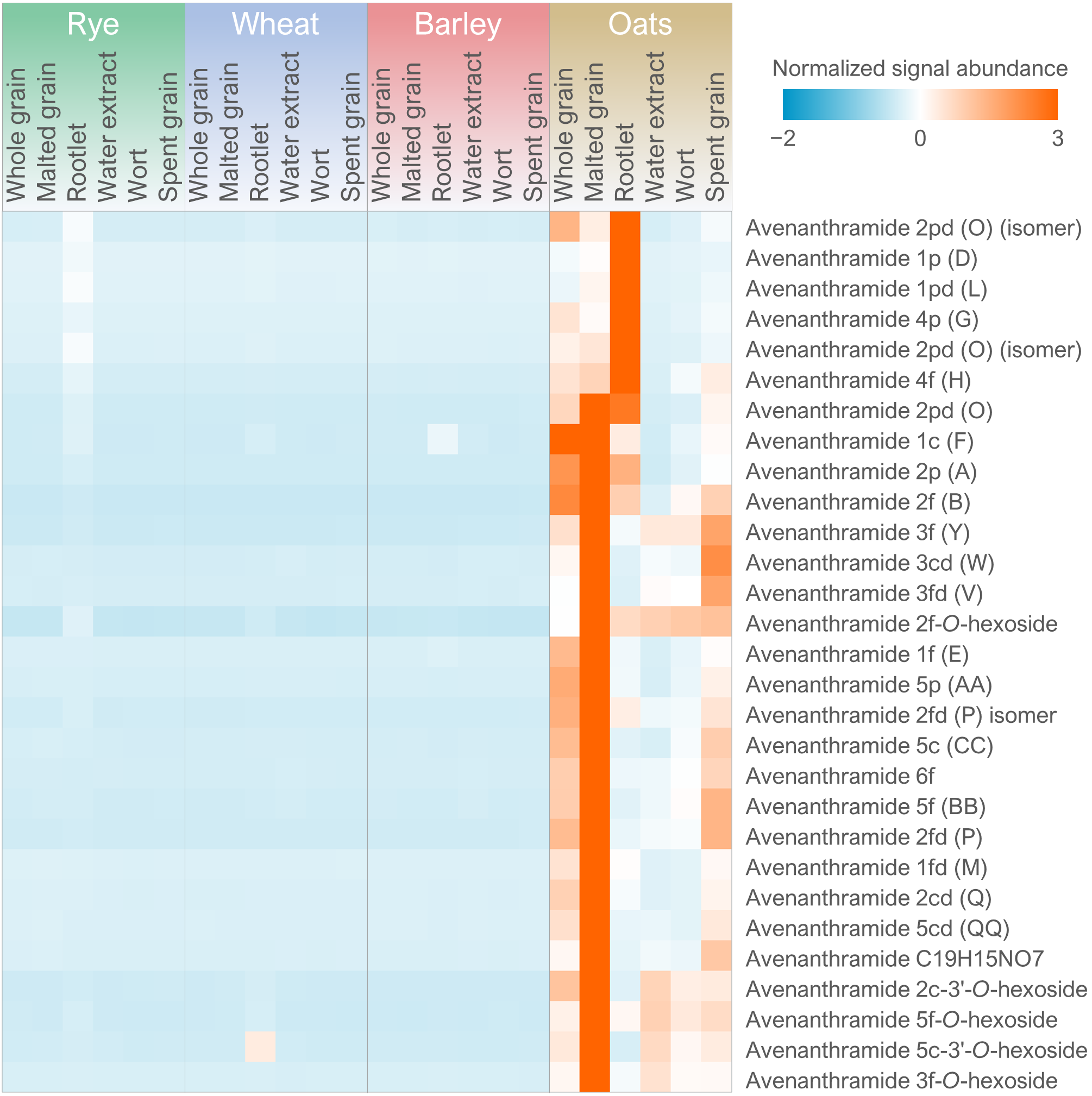


**C) Benzoxazinoids**

**
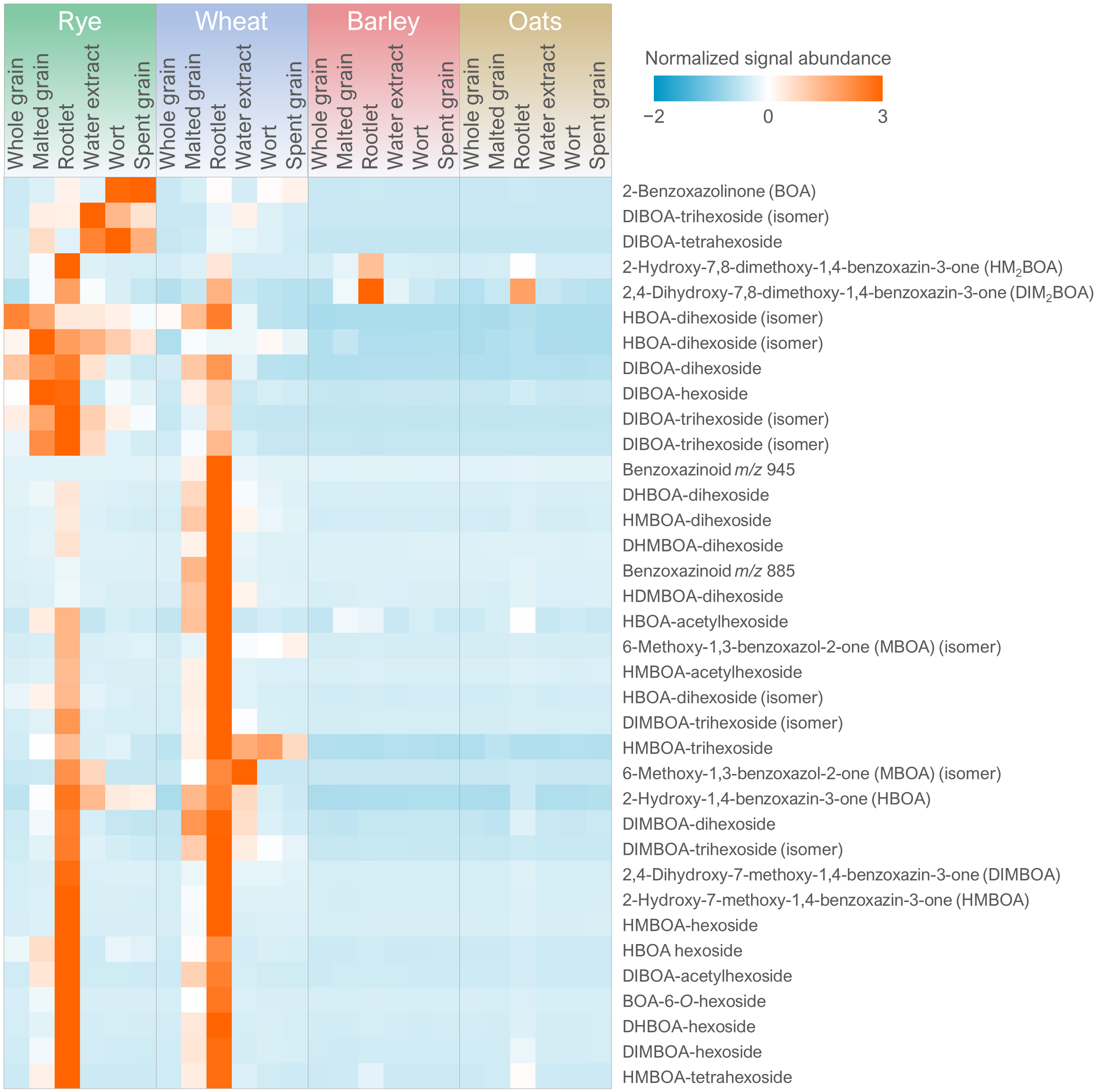
**

**D) Flavonoids**

**
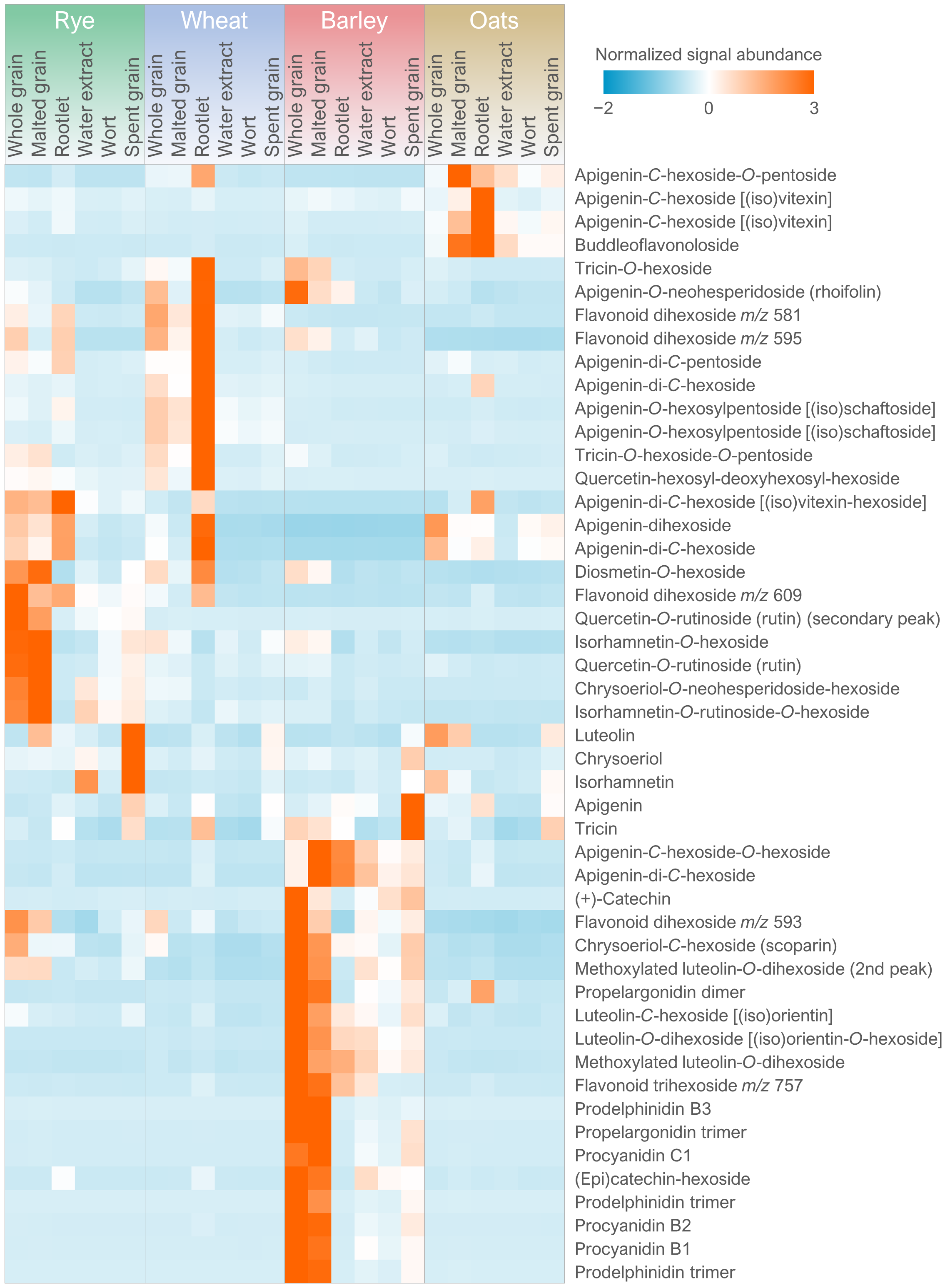
**

**E) Lignans**

**
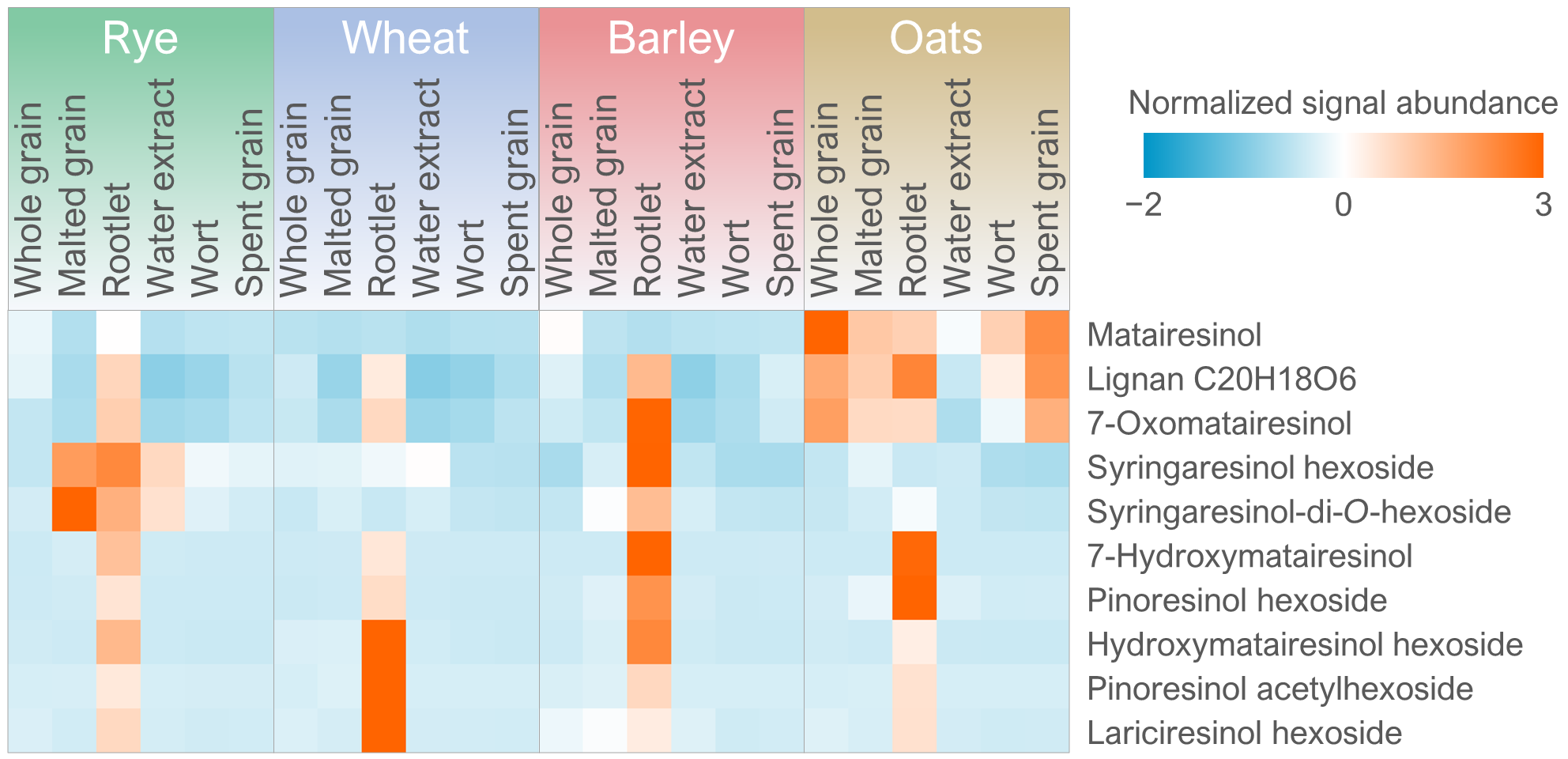
**

**F) Phenolamides**

**
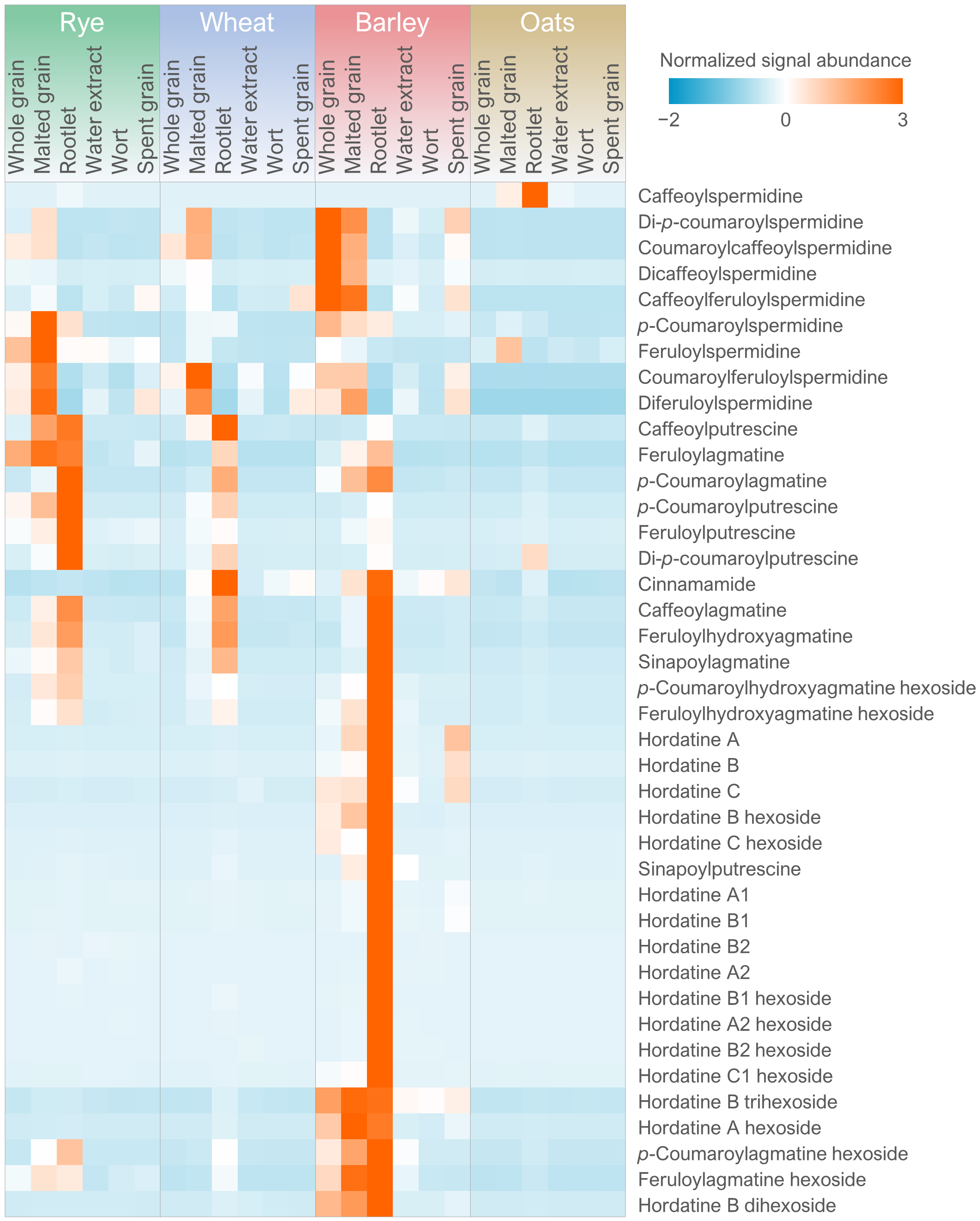
**

**G) Phenolic acids**

**
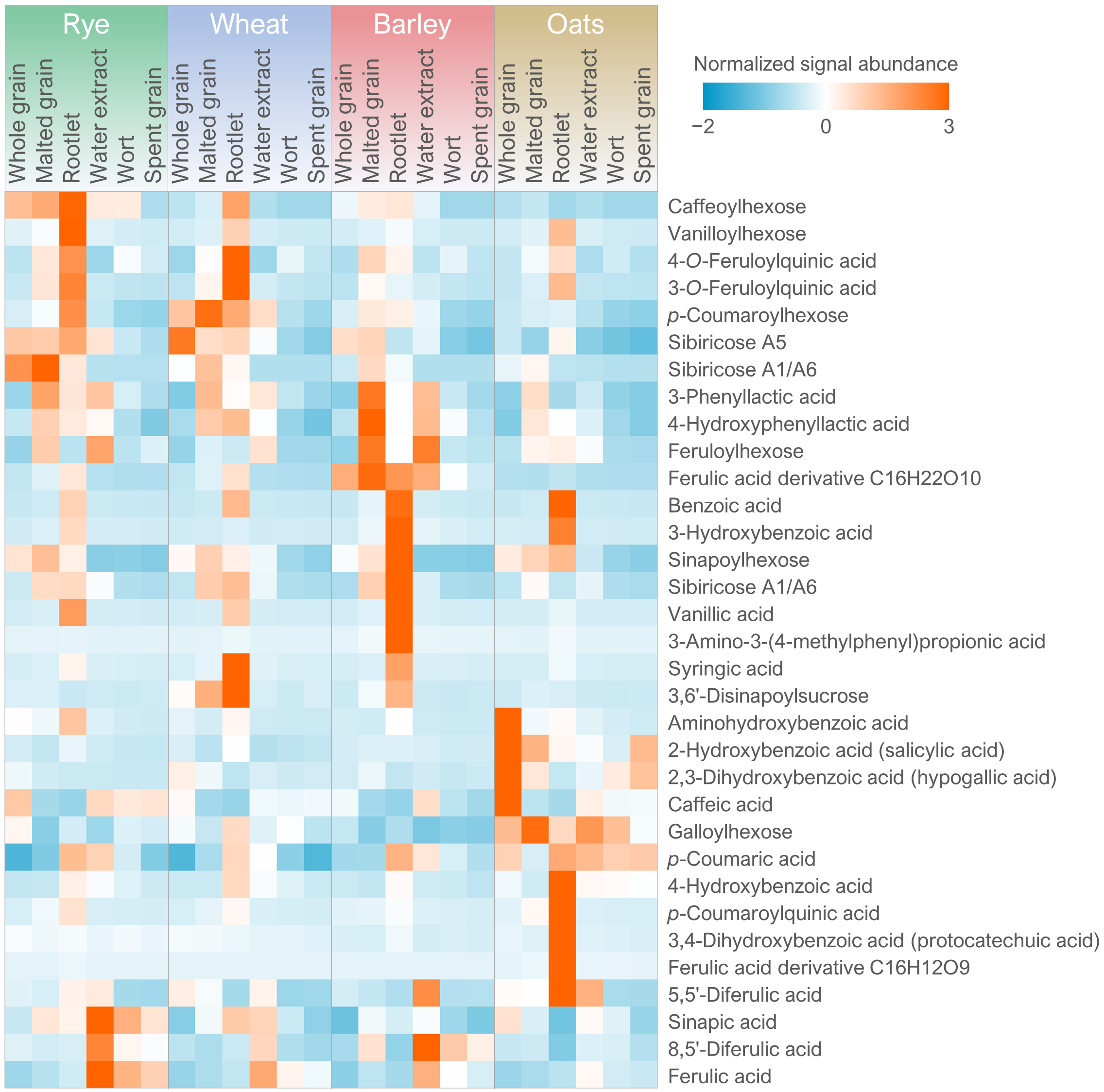
**

**H) Phytosterols**

**
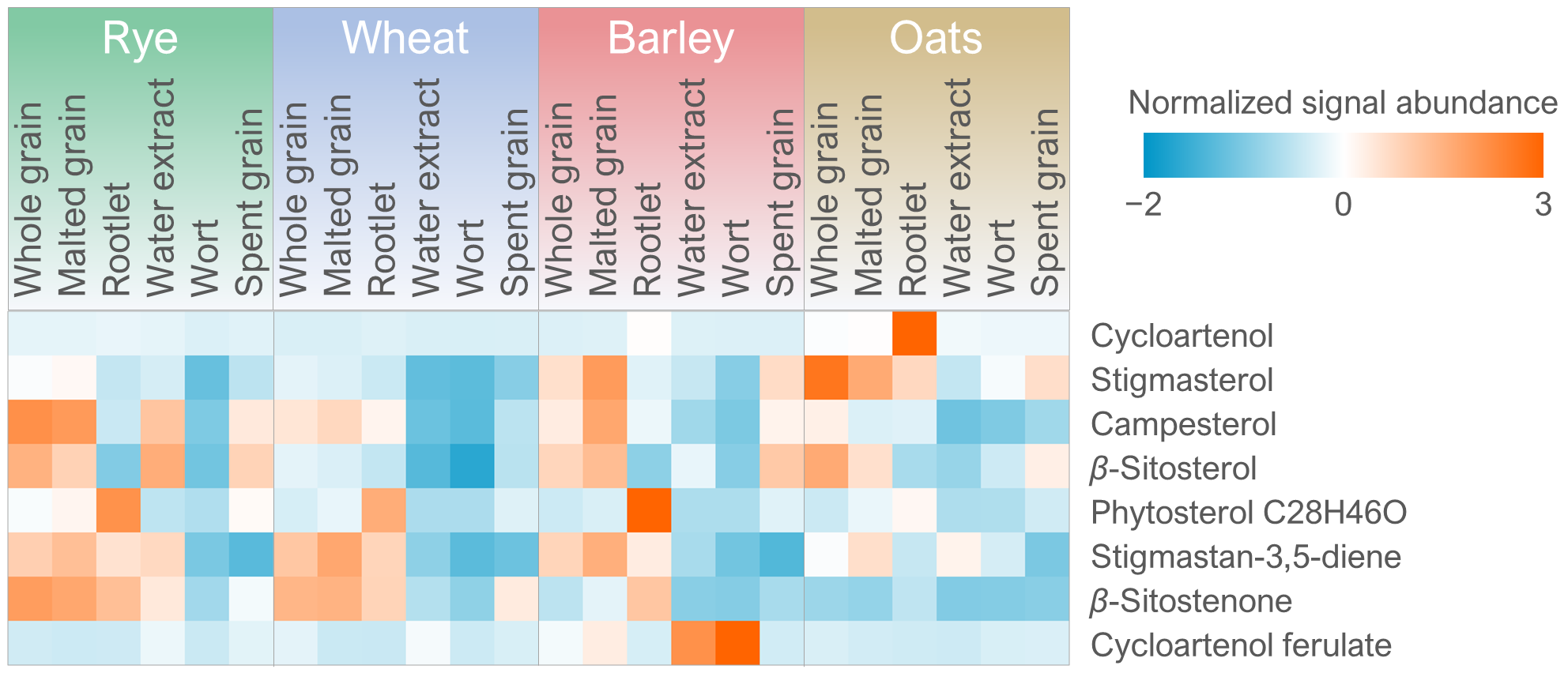
**

**I) Saponins**

**
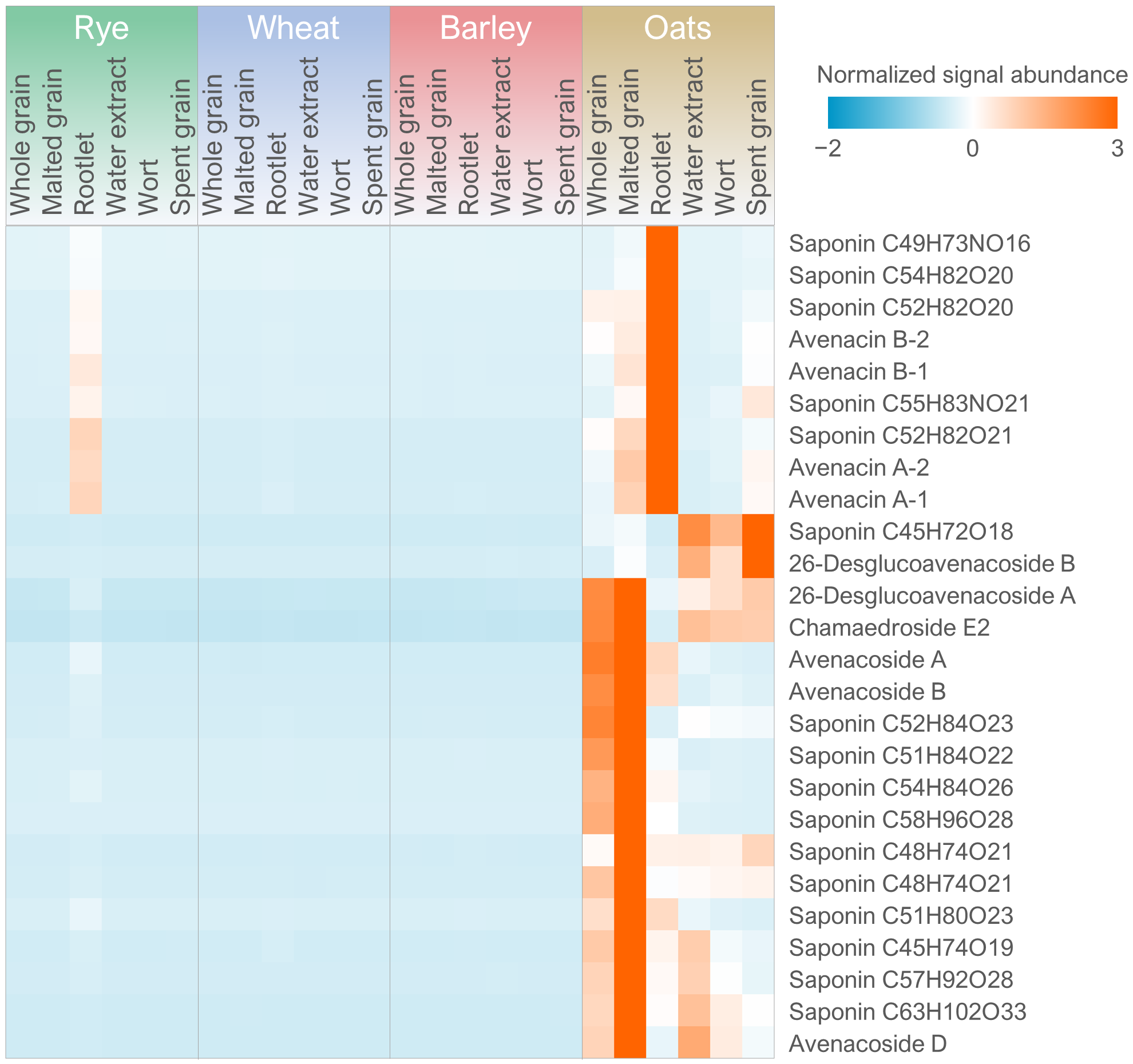
**

**J) Tocols**

**
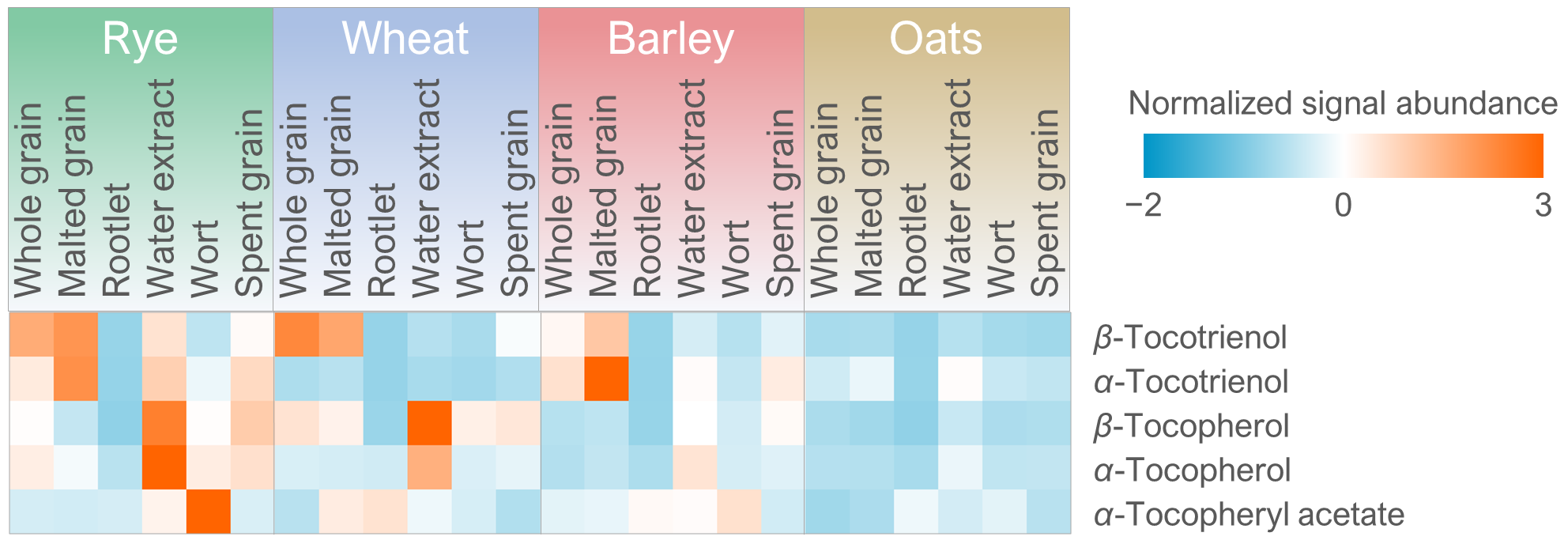
**

**K) Other phytochemicals**

**
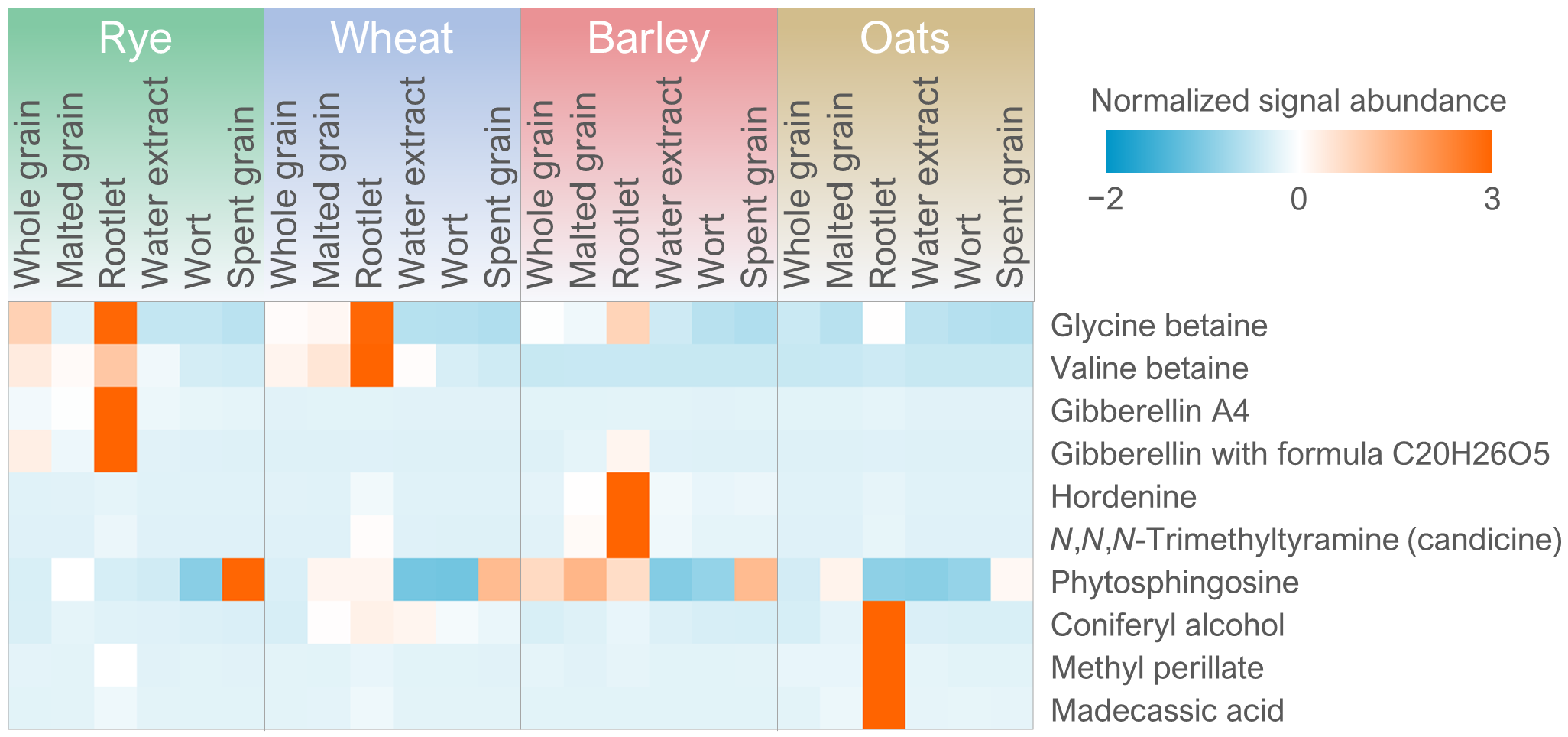
**
